# Restrictive approach to episiotomy and the risk of third- and fourth-grade perineal tears: An analytical cross-sectional study at a rural tertiary hospital in South Africa

**DOI:** 10.4102/jcmsa.v4i1.242

**Published:** 2026-02-17

**Authors:** Masibonge S. Ndlela, Charles B. Businge

**Affiliations:** 1 Department of Obstetrics and Gynaecology, Faculty of Medicine and Health Sciences, Walter Sisulu University, Mthatha, South Africa; 2 Department of Obstetrics and Gynaecology, Mthatha Regional Hospital, Mthatha, South Africa

**Keywords:** episiotomy rate, restrictive episiotomy, gravidity, maternal age, perineal tears, analytical cross-sectional study, Eastern Cape

## Abstract

**Background:**

Based on current evidence, the World Health Organization (WHO) recommends a restrictive approach to episiotomy, rather than routine performance, with a rate of between 5% and 10%. This study aimed to ascertain the rate and conformity to the restrictive patterns of episiotomy among women delivering at Mthatha Regional Hospital (MRH).

**Methods:**

A cross-sectional analytical study was conducted with 400 participants at MRH between January and June 2022. Demographic and obstetric data were collected and analysed using SPSS software. Continuous data were analysed using the student’s *t*-test for normally distributed data and the Wilcoxon–Mann–Whitney *U*-test for skewed data, categorical data with the chi-square and Fisher’s tests, and logistic regression for the independent predictors of episiotomy. A *p*-value < 0.05 was considered significant.

**Results:**

The rate of episiotomy was 19% (74/400). The indications for episiotomy were macrosomia (8.1%), foetal distress during the second stage of labour (5.4%), delayed second stage of labour (5.4%), prolonged second stage (5.4%), previous C/S x2 in labour (2.7%), forceps delivery (2.7%) and tight perineum (1.3%); 74% (55/74) was undocumented. The factors associated with episiotomy were maternal age < 20 years (odds ratio [OR] 5.5, confidence interval [CI] 3.2–9.5) and primigravid status (OR 10.2, CI 5.2 – 19), *p* < 0.05. The rate of third-degree perineal tears among women without episiotomy was 0.3%. There were no fourth-degree tears.

**Conclusion:**

The practice of episiotomy at MRH exceeds the recommendation by the WHO. Young maternal age and primigravid women were risk factors for episiotomy.

**Contribution:**

The low incidence of third- and fourth-degree tears among women without episiotomy reaffirms the advantages of a restrictive approach to the practice of episiotomy.

## Introduction

### Background

Episiotomy is the most common surgical procedure performed by midwives and obstetricians in the second stage of labour. It is defined as a perineum enlargement incision during the second stage of labour to increase the diameter of the vaginal outlet to facilitate baby’s birth.^1^ It was first reported in 1742 and deemed an emergency procedure, only to be carried out for the benefit of the baby.^2^ The shift to being performed routinely occurred in the 1800s, with potential benefits being cited as prevention of laceration, shortening of labour and prevention of gynaecological prolapse in the future.^3,4^ A systematic review demonstrated that episiotomy did not protect from perineal trauma, asphyxia or foetal intracranial haemorrhage.^5^ Routine episiotomy exposed women to complications such as wound site oedema, wound site infection, sexual dysfunction and anorectal dysfunction, including incontinence.^6,7^

The World Health Organization (WHO) recommends an indication-based, restrictive use of episiotomy, with rates of less than or equal to 10%.^8^ Implementation of the restrictive use of episiotomy is associated with up to a 65% reduction in the need for episiotomy.^9^

Globally, the prevalence of episiotomy varies, with Denmark at 4.9%, Sweden at 9.7% and France at 14.3%. It is reported in India to be 68%, and in Taiwan, 100%.^10,11^ The overall rate of episiotomy in Africa is 41.7%. In Uganda, it is about 73%, Ethiopia 68%, Burkina Faso 21%, Ghana 17%, Nigeria 9.3% and South Africa 63.3%.^1,11^

Recent studies have tried to investigate the disparity and have identified several factors. Nulliparity was associated with an episiotomy rate of 64% compared to 27% in multiparous women; gestational age of greater than 41 weeks had an episiotomy risk that is 1.5 times higher; foetal weight of greater than 4000 g increases the rate of episiotomy by five times.^12,13^ Others are delayed second stage, especially beyond 2 h, which increases the episiotomy risk by 4.6 times, and assisted delivery, especially with forceps, which increases the risk by 3.9 times.^11,12^

Although South African guidelines advocate for the restrictive use of episiotomy, this is still not the case in some regions of the country. Routine episiotomy is still a common practice, with 35.4% of midwives and 44.4% of obstetricians agreeing that episiotomies should be performed routinely at every birth.^14^ Reasons cited for the persistent use of routine episiotomy were to expedite delivery (51% of practitioners) and concerns of third- and fourth-degree tears.^14^

Restrictive episiotomy is associated with a 30% reduction in severe perineal or vaginal trauma among both primiparous and multiparous women, a 9% less need for perineal wound suture and a 15% drop in moderate to severe pain in the postpartum period.^15,16^

Women with restrictive episiotomy and routine episiotomy share similar outcomes with regard to local wound complications, birth asphyxia and neonatal intensive care unit (NICU) admissions. There is no documented difference in intrapartum blood loss and rates of post postpartum haemorrhage (PPH).^15^

It is not yet established whether evidence-based use of restrictive episiotomy has been implemented in medical facilities in the Eastern Cape. Wide implementation of restrictive use of episiotomy in the delivery units is associated with up to a 65% reduction in the need for episiotomy and fewer perineal tears.

The aim of the study was to ascertain the prevalence of episiotomy among women delivering at Mthatha Regional Hospital (MRH) and whether the indications conformed to the restrictive patterns advocated by the WHO, as well as to establish the rate of third- and fourth-degree perineal tears among women with and without episiotomy.

## Research methods and design

### Study design

This was a hospital-based cross-sectional study that involved a retrospective record review of women who delivered at MRH between January 2022 and June 2022.

### Study setting

The study was conducted at MRH, situated in Mthatha, the main town of the King Sabata Dalindyebo Municipality in the Eastern Cape. It is a 302-bed hospital with an average of 560 deliveries monthly. It offers level 1 and level 2 services and receives referrals from Mbekweni, Baziya and Ngangelizwe community health centres.

### Sample size calculation

We determined the sample size using the formula (Equation 1):


$$N=Z^{2}P(1-P)/d^{2}$$ [Eqn 1]

where *N* is the sample size, *Z* is the statistic at the 5% level of significance, *P* = 0.63, that is, the rate of episiotomy reported in a previous South African study,^1^ and *d* is the precision or the effect size, taken here as 5%.

Taking into consideration that 10% of the sample might have missing information, the sample size was 398.

### Inclusion criteria

All medical records of the women who delivered via vaginal delivery at MRH from January 2022 to June 2022 were eligible for inclusion in the study.

### Exclusion criteria

The records of women who had caesarean delivery at MRH during the study period were excluded.

### Sampling method

Systematic random sampling of 100 cases of vaginal deliveries per month was used. Every third vaginal delivery’s folder was selected until 100 case records of women who had vaginal delivery were obtained for each of the 4 months from January 2022 to April 2022.

### Data collection and management

Data were collected from the maternity case charts and labour ward records using uniquely coded sheets with study numbers. The variables recorded included the chronological age, gravidity, parity, gestational age, HIV status and viral suppression at the time of delivery, cadre of the health worker that conducted the delivery, duration of the second stage of labour, the indication for the episiotomy, the need for assisted delivery, foetal head circumference and birthweight.

### Data analysis

Data were doublechecked for accuracy and entered in an Excel spreadsheet and then imported into SPSS (IBM Version V.29. 2022) for statistical analysis. Categorical data were expressed as frequencies and proportions (with 95% confidence intervals [CI]); continuous variables were presented as means ± standard deviation (s.d.) if normally distributed and as median (Interquartile Range [IQR]) if not normally distributed. The chi-square test, Mann–Whitney and binary regression were used to make comparisons between women with and without particular attributes. Both univariate and multivariate analyses were carried out to identify independent risk factors for maternal and neonatal adverse outcomes. Statistical significance level was set at 5% (*p* < 0.05).

### Ethical considerations

Ethical approval and waiver of consent were sought and obtained from the Human Research Ethics Committee of Walter Sisulu University (WSU HREC No. 134/2024). The study was conducted in keeping with the Helsinki Declaration.

## Results

A sample of 400 medical records out of a total of 2200 belonging to the women who delivered at the study site during the study period was obtained. Of these 400 women, 114 (29%) were in the age group of 20–24 years, 104 (26%) were in the age group of 25–29 years, 40 (10%) were over 35 years and the minority, 6 (2%), were in the age group of < 15 years. Two hundred and forty-four (61%) were multigravidae and 156 (39%) were primigravidae. Almost three-quarters, that is, 294 (74%) of the participants were between 37 weeks and 42 weeks of gestation (Table 1).

**TABLE 1:** Characteristics of the participants (*N* = 400).

Variable	*n*	%
**Age (years)**		
< 15	6	1.5
15–19	81	20.2
20–24	116	29.0
25–29	102	25.5
30–34	55	13.8
35+	40	10.0
**Gravidity**		
Multipara	244	61.0
Primigravida	156	39.0
**Gestation age**		
< 37 WOA	106	26.6
37–42 WOA	292	73.4

WOA, weeks of amenorrhoea.

### Birth-related characteristics

Most mothers, 394 (98.5%), were delivered by midwives, 4 (1%) by medical officers and 2 (0.5%) by consultant obstetricians. The majority of the participants, 396 (99%), had a normal vertex delivery, and the remaining 4 (1%) had assisted vaginal deliveries. Almost 90% (359/400, 89.8%) of participants delivered babies with a birthweight ranging from 2.5 kg to 3.9 kg, followed by 27 (7%) between 1.5 kg and 2.4 kg, 13 (3%) more than 4 kg, while only one was less than 1 kg. More than half of the mothers (213/398, 53%) delivered babies with head circumference ranging between 35 cm and 40 cm (Table 2).

**TABLE 2:** Birth-related characteristics of the participants (*N* = 400).

Variable	*n*	%
**Birth attendant**		
Consultant	2	0.5
Medical officer	4	1.0
Midwife	394	98.5
**Duration of the second stage (h)**		
< 1	357	89.9
1–2	28	7.1
> 2	12	3.0
**Frequency of episiotomy**		
Episiotomy	74	18.5
No episiotomy	326	81.5
**Perineal tear**		
Intact	178	44.6
First-degree tear	124	31.1
Second-degree tear	22	5.5
Third-degree tear	1	0.3
No record	1	0.3
**Assisted delivery**		
Forceps	2	0.5
Vacuum	2	0.5
Not applicable	396	99
**Birthweight (kg)**		
< 1	1	0.2
1.5–2.4	26	6.5
2.5–3.9	360	90.0
> 4	13	3.2
**Head circumference (cm)**		
< 35	187	46.8
35–40	213	53.2
**Indication for episiotomy**		
Undocumented	55	74.3
Delayed second stage	4	5.4
Foetal distress in second stage	2	2.7
Forceps delivery	2	2.7
Vacuum	2	2.7
Macrosomia	6	8.1
Prev. C/S x 2 in labour	2	2.7
Tight perineum	1	1.3

Prev., previous; C/S, caesarean section.

Seventy-four of the 400 mothers (19%) had an episiotomy. Of these 74 participants who had an episiotomy, 55 (74.3%) had no specified indication, 6 (8.1) were done for macrosomia, 4 (5.4%) for foetal distress during second stage of labour, 4 (5.4%) for delayed second stage of labour, 2 (2.7%) for previous c/s x 2 in labour, 2 (2.7%) for forceps delivery, 4 (5.4%) for prolonged second stage and 1(1.3%) for a tight perineum.

Of the 324 mothers who delivered without an episiotomy, 178 (45%) had an intact perineum, 124 (31%) had a first-degree perineal tear, 22 (5%) had a second-degree perineal tear and 1 (0.3%) had a third-degree perineal tear. There were no fourth-degree perineal tears.

### Factors associated with an increased frequency of episiotomy

The demographics and birth-related variables of participants with and without episiotomy were compared (Table 3, Table 4, and Table 5). Maternal age below 20 years, primigravid status, a positive HIV status, delivery conducted by a doctor and second stage duration of 1–2 h were significantly associated with the risk of episiotomy.

**TABLE 3:** Odds of episiotomy according to maternal age, gravidity and gestational age at delivery.

Variable	Episiotomy	No episiotomy	OR	CI	*p*-value
**Age (years)**	-	-	5.5	3.2–9.5	< 0.0001
< 20	37	50	-	-	-
21–49	37	276	-	-	-
**Gravidity**	-	-	10.2	5.5–19.0	< 0.0001
Primigravida	60	96	-	-	-
Multigravida	14	230	-	-	
**Gestation age (WOA)**	-	-	0.9	0.48–1.55	0.6187
< 37	18	88	-	-	-
37–42	56	236	-	-	-

WOA, weeks of amenorrhoea; OR, odds ratio; CI, confidence interval.

**TABLE 4:** Odds of episiotomy according to human immunodeficiency virus status and viral load.

Variable	*n*	Episiotomy	No episiotomy	OR	CI	*p*-value
**HIV status**	-	-	-	0.31	0.15–0.68	0.0032
Negative	301	66	235	-	-	-
Positive	99	8	91	-	-	-
**HIV viral load**	-	-	-	0.36	0.08–1.6	0.1774
Suppressed	60	3	57	-	-	-
Unsuppressed	39	5	34	-	-	-

OR, odds ratio; CI, confidence interval.

**TABLE 5:** Birth-related factors associated with episiotomy (*N* = 400).

Variable	Episiotomy		No episiotomy		Chi-square	*p*-value
	*n*	%	*n*	%		
**Birth attendant**	-	-	-	-	9.564*	0.001
Consultant	2	2.7	0	0.0	-	-
Medical officer	3	4.1	1	0.3	-	-
Midwife	69	93.2	325	99.7	-	-
**Duration of second stage (h)**	-	-	-	-	4.758*	0.09
< 1	63	85.1	294	91.0	-	-
1–2	10	13.5	18	5.6	-	-
> 2	1	1.4	11	3.4	-	-
**Assisted delivery**					-	-
Forceps	2	2.7	0	0.0	-	-
Vacuum	2	2.7	0	0.0	-	-
**Birthweight (kg)**	-	-	-	-	0.7020*	0.8727
< 1	0	0.0	1	0.3	-	-
1.5–2.4	4	5.4	22	6.7	-	-
2.5–3.9	68	91.9	292	89.6	-	-
> 4	2	2.7	11	3.4	-	-
**Head circumference (cm)**	-	-	-	-	0.24868	0.618
< 35	32	43.8	152	47.1	-	-
35–40	41	56.2	171	52.9	-	-

* , Denotes Yates’ chi-square.

The proportion of women delivered by obstetricians and medical officers was higher among participants who received an episiotomy (6.8%) than those without (0.3%) (Yates’ *p* < 0.001). Although not statistically significant, the proportion of participants with the duration of the second stage > 1 h was higher among women who received an episiotomy (15%) than those who did not (9%) (Yates’ *p* = 0.090). All four participants who required assisted delivery had an episiotomy. Birthweight and head circumference were not significantly associated with episiotomy (Table 5).

### Independent predictors of episiotomy

The variables significantly associated with episiotomy and the duration of the second stage that showed a higher trend among women with episiotomy were entered into logistic regression models to determine adjusted ORs, the 95% CIs and *p*-values (Table 6). Age and gravidity were entered into Model 1. Model 2 comprised the variables in Model 1 in addition to the HIV status. Model 3 comprised all the birth-related predictors that, on univariable analysis, were significantly associated with episiotomy.

**TABLE 6:** Logistic regression models for predictors of episiotomy.

Predictors	Model 1 (Age, Gravidity)			Model 2 (Age, Gravidity, RVD)			Model 3 (Age, Gravidity, Birth Attendant, Duration of Second Stage)		
	AOR	95% CI	*p*-value	AOR	95% CI	*p*-value	AOR	95% CI	*p*-value
**Age (years)**									
< 20	1	-	-	1	-	-	1	-	-
20–24	0.40	0.18–0.82	0.012*	0.41	0.20–0.84	0.015*	0.39	0.19–0.83	0.014*
25–29	0.66	0.28–1.57	0.351	0.72	0.30–1.74	0.467	0.64	0.26–1.55	0.319
30+	0.86	0.30–2.45	0.782	0.96	0.33–2.77	0.940	0.84	0.29–2.43	0.747
**Gravidity**									
Multipara	1	-	-	1	-	-	1	-	-
Primigravida	9.87	4.40–22.13	< 0.001***	9.43	4.21–21.16	< 0.001***	9.09	4.00–20.64	< 0.001***

Note: Significant predictors are shown as:

* *p* < 0.05;

*** *p* < 0.001.

AOR, adjusted odds ratio; CI, confidence interval; RVD, retro-viral disease.

The results of these models indicate that gravidity is the most significant predictor of episiotomy, with first-time mothers having 9 to 10 times the odds of receiving an episiotomy than experienced mothers. Similarly, mothers aged 20 years and older have lower odds of an episiotomy than those under 20 years. The addition of HIV status and professional category of the personnel who conducted the delivery to Model 2 did not significantly alter the odds of episiotomy. Similarly, the inclusion of the duration of the second stage had no significant change on the odds of episiotomy.

## Discussion

### Prevalence of episiotomy

This study found an episiotomy rate of 19% among participants. This is higher than the rate proposed by the WHO of 10%, which is achievable through a restrictive rather than liberal episiotomy.^17^ The episiotomy rate established from this study is, however, lower than the overall rate in South Africa, which is 63.3% and in Africa (41%).^18^

Globally, there is a disparity in the rates, with Denmark, 4.9%, the United States, 9.4%, the United Kingdom, 15.2% in 2011/2012 and France, 14.3%.^11^ The episiotomy rate in most African nations is still higher than the WHO recommendation: Rwanda at 80%, Uganda at 73%, Ethiopia at 68%, Zimbabwe at 26% and Burkina Faso at 21%. Nigeria has the lowest rate at 9.5%, meeting the WHO standards.^11,12,18^ The disparity may partially be attributed to the difference in the knowledge gap of skilled birth attendants, the use of institutional protocol adapted from the restrictive approach advocated by the WHO, the availability of ongoing training of birth attendants, the level of institution, the different services offered and the practice of evidence-based medicine.

### Indications of episiotomy

Of the 74 participants who had an episiotomy, 19 (25.6%) had indications that aligned with the restrictive practice advocated by the WHO and the South African National Department of Health. The highest indication was foetal macrosomia (8.1%), delayed second stage at 5.4%, with the lowest being tight perineum at 1.3%. This study differs from a study conducted in Nigeria in 2021 that reported that a tight perineum accounted for more than half of all episiotomies, followed by shoulder dystocia, instrumental delivery and lastly breech.^19^ In this study, 55 participants (74.3%) had no documented indication of episiotomy. This could account for the difference in the indications noted in our study and the above-mentioned study.

The high percentage of undocumented indication of episiotomy may have been because of routinely conducted episiotomies without consideration of the restrictive approach advocated by the WHO. Furthermore, it may partially be accounted for by the lack of a designated area to record the indication in the type of maternity case records currently in use in the Eastern Cape. As this was a retrospective study, it was not possible to establish if the midwives and doctors who conducted the deliveries were up to date with the latest literature concerning restrictive episiotomy practice. Hence, a follow-up study is needed to establish the knowledge and attitude of the health workers in the study area about routine and restrictive episiotomy, including the advantages and adverse consequences.

### Factors associated with episiotomy

In this study, the independent predictors of episiotomy were maternal age lower than 20 years, primigravidity and delayed second stage of labour. This is in keeping with recent studies from Poland and Iran that also reported a similar association between maternal age, parity and the risk of episiotomy.^20,21^

This was further echoed in a study from southern Brazil that reported a higher rate of episiotomy among adolescents compared to mothers older than 35 years.^22^ In another study from Ethiopia, the authors found that mothers between the ages of 25 years and 35 years were 89% less likely to have an episiotomy than those aged 18 years to 24 years.^18^

Furthermore, 81% of the participants in this study who received an episiotomy were primigravidae.

This is similar to the results found in one study from Iran that showed that 90% of the participants who received an episiotomy were primigravidae.^21^ This is, however, much higher than the rate reported by Woldegeorgis et al. of 64% among primigravidae compared to that of multipara at 27%, although the trend is similar.^11^

The primigravidity status in this study conferred a 9 to 10 odds for receiving an episiotomy. Our results are in keeping with studies in Ethiopia and Nigeria^23,24^ but twice as high as the risk of 4.79 in the study reported by Deyaso et al.^25^ This could be attributed to the persistent practice of a routine, non-restrictive approach to episiotomy when delivering primigravidae in anticipation of a tight perineum.^1,25^

In this study, the duration of the second stage of labour was longer among women who received an episiotomy compared to those without an episiotomy (duration of second stage > 1 h 15% vs 9%). Worku et al. similarly demonstrated a positive association between episiotomy risk and prolonged second stage of more than 2 h.^1^ The odds of episiotomy following a prolonged second stage of more than 2 h range between 4.6 and 9.3.^11,25^ Although the odds of episiotomy associated with prolonged second stage of labour in this study were 1.54, this was not statistically significant (*p* = 0.267).

In this study, 5.4% (4/74) of the participants had an episiotomy to facilitate assisted vaginal delivery: two were assisted with a vacuum, and the other two with forceps. None of the 326 participants without an episiotomy had a vacuum or forceps delivery. Assisted vaginal delivery is one of the indications for restricted episiotomy and may explain this result in the current study and those of previous studies. A study from Metema district, northwest of Addis Ababa, reported that the likelihood of an episiotomy among women with assisted vaginal delivery was about 3.04.^18^ Furthermore, Deyaso et al., in a systematic review and meta-analysis of the risk factors of episiotomy in Ethiopia, reported that women undergoing assisted vaginal delivery were more than four times likely to have an episiotomy than those without assisted vaginal delivery.^25^

### HIV status and the risk of episiotomy

The HIV status in this study was significantly associated with reduced odds of episiotomy. This is attributed to the earlier studies that found that episiotomy was associated with increased risk of mother-to-child transmission of HIV, which informs current practice that discourages routine episiotomy for HIV-positive mothers.^26,27,28^ Although there was a trend towards reduced odds of episiotomy among women with unsuppressed HIV viral load, this was not statistically significant, probably because of inadequate sample size with insufficient power to test this association. Current evidence suggests that women with virally suppressed HIV can safely have an episiotomy and instrumental vaginal delivery without increased risk of maternal-to-child transmission of HIV. Caesarean delivery is recommended for those with unsuppressed HIV viral load.^29^

### Association of the risk of episiotomy with the cadre of health personnel conducting the delivery

More than 90% (93%) of the episiotomies in this study were performed by midwives, 2.7% by consultants and 4.1% by medical officers. This is similar to a study from Nigeria in which 75% of the deliveries were performed by midwives.^30^ This is because most of the women delivering at MRH are low risk. Hence, the majority of the deliveries in this study (98.5%) were conducted by midwives, 1% by medical officers and 0.5% by consultants.

Irrespective of the cadre of skilled birth attendants (whether midwives, medical officers or specialists), limited knowledge about the evidence-based practice of restrictive episiotomy and a lack of institutional guidelines on the practice of episiotomy are associated with high rates much above the recommendation of the WHO. Similar findings were reported in a recent study carried out in KwaZulu-Natal province of South Africa, which has many similarities with the site of this study.^14^ Furthermore, clinicians who completed formal training much earlier had a higher propensity to perform an episiotomy and were more inclined to stick to the routine practice of episiotomy than their recently qualified counterparts.^31,32^ These factors could not be evaluated with the data in this retrospective study. Hence, a follow-up study is required to assess how age, level of qualification, work experience and in-service continuous medical education influence the practice of episiotomy in the study setting.

### Rate of third- and fourth-degree perineal tears

Of the 325 participants who had spontaneous vaginal delivery without episiotomy, only 1 (0.3%) sustained a third-degree perineal tear. There were no participants with fourth-degree tears. This reaffirms that vaginal delivery without routine episiotomy does not increase the risk of third- and fourth-degree perineal tears.^5,16^ Instead, a restrictive approach to the practice of episiotomy helps prevent the short-term and long-term complications associated with episiotomy, such as damage to the perineal body and anal sphincter, faecal incontinence, postpartum pain and dyspareunia.^21,33^

### Study limitations

This study was conducted at a regional hospital; therefore, the results cannot be freely generalised to the district hospitals and health centres that perform the bulk of deliveries in the Eastern Cape. Being a retrospective review of the medical records, some information was missing. A prospective study would have given a better insight into the practice of episiotomy in the study area, as well as collected data regarding the attitude of skilled birth attendants towards restrictive episiotomy practice. Furthermore, the exclusion of all women who had a caesarean section also excluded women who had an episiotomy and then had emergency caesarean delivery for prolonged second stage, which could have created some bias.

## Conclusion

This study showed a higher episiotomy practice in MRH in comparison to the WHO recommendation. It showed that young maternal age and primiparity were risk factors for episiotomy. The low incidence of third- and fourth-degree tears among women without episiotomy reaffirms the advantages of a restrictive approach to the practice of episiotomy. It is prudent to conduct regular in-service training, and continuous medical education so as to encourage adherence to evidence-based practices such a restrictive approach to episiotomy. It is also necessary to modify the current antenatal card to provide a space for the documentation of the indication of episiotomy.
